# Retraction: Pleiotropic role of Drosophila *phosphoribosyl pyrophosphate synthetase* in autophagy and lysosome homeostasis

**DOI:** 10.1371/journal.pgen.1010048

**Published:** 2022-02-04

**Authors:** Keemo Delos Santos, Minhee Kim, Christine Yergeau, Steve Jean, Nam-Sung Moon

Following the publication of this article [1], the corresponding author contacted *PLOS Genetics* to indicate that it has come to their attention that the flies used in this study did not carry the patient derived PRPS mutations described in the article. The corresponding author clarified that in light of their recent findings the results and conclusions reported in this study are unreliable, and requested retraction of the article.

All authors agree with the retraction and apologize for the issues with the published article.
